# The Clinical Course and Management of Severe Esophagitis Dissecans Superficialis: A Case Report

**DOI:** 10.1177/2324709619892726

**Published:** 2019-12-10

**Authors:** Isaac Jaben, Richard Schatz, Ira Willner

**Affiliations:** 1 Medical University of South Carolina, Charleston, SC, USA

**Keywords:** esophagus, endoscopy, dysphagia, odynophagia

## Abstract

Esophagitis dissecans superficialis is a rare clinical endoscopic finding with poorly understood pathogenesis and ill-defined management. A 71-year-old man is admitted with progressively worsening dysphagia and odynophagia with endoscopic features most consistent with severe esophagitis dissecans superficialis. Extensive workup did not reveal an etiology, and he was subsequently treated with steroids, resulting in rapid, almost complete clinical and endoscopic recovery.

## Introduction

A rare yet dramatic endoscopic finding, esophagitis dissecans superficialis (EDS) can be an incidental discovery or cause debilitating symptoms. Frequently presenting with vague symptoms and prolonged progression of disease, EDS can easily be misidentified or unrecognized due to its frequently vague presenting symptoms and gradual progression. Limited knowledge regarding pathogenesis and treatment of this condition complicates management. Using an especially severe case, we discuss the presentation, pathogenesis, and management of EDS.

## Case Report

**Figure 1.**
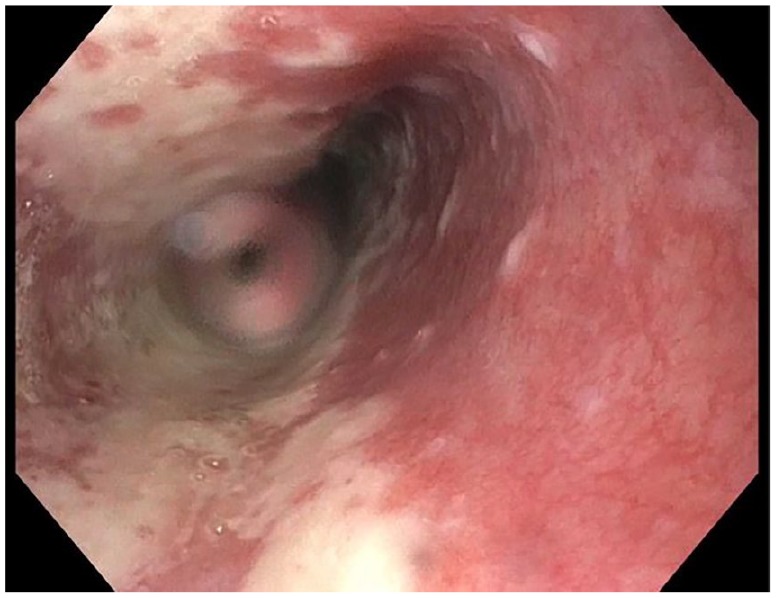
Severe esophagitis of the mid-esophagus seen on initial esophagogastroduodenoscopy.

The case is of a 71-year-old man with a history of coronary artery disease status post coronary artery bypass grafting, hypertension, and type 2 diabetes mellitus, who initially was evaluated in the outpatient gastroenterology clinic for progressively chest pain, regurgitation, and mild odynophagia. His symptoms had developed over the course of several months and were initially felt to be due to uncontrolled, severe gastroesophageal reflux. Esophagogastroduodenoscopy (EGD) was performed, and it revealed severe esophagitis (Figure 1). Multiple biopsies taken of the affected areas remarked only upon sloughed esophageal mucosa. Outpatient treatment with a twice-daily, high-dose oral proton pump inhibitor for 2 months improved most symptoms. Repeat EGD, 2 months later, demonstrated a benign, distal esophageal stricture that was dilated with good endoscopic and symptomatic effect. Over the next year, his symptoms gradually returned. He regurgitated almost all solid foods and also had new, intractable nausea culminating in 2 weeks of anorexia with associated 10-pound weight loss. He was then admitted to the hospital for further evaluation and management.

**Figure 2.**
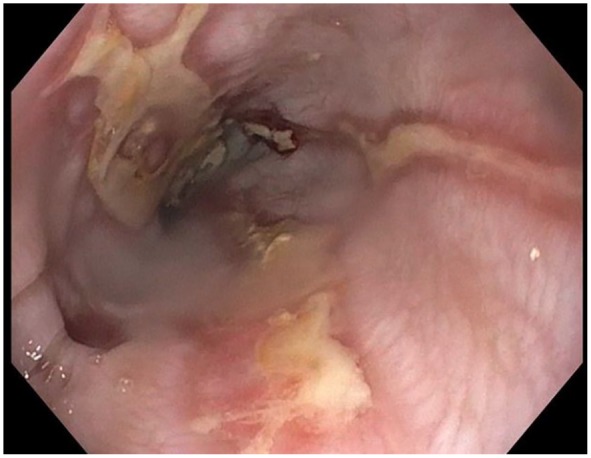
Progression of disease with characteristic sloughing mucosa.

**Figure 3.**
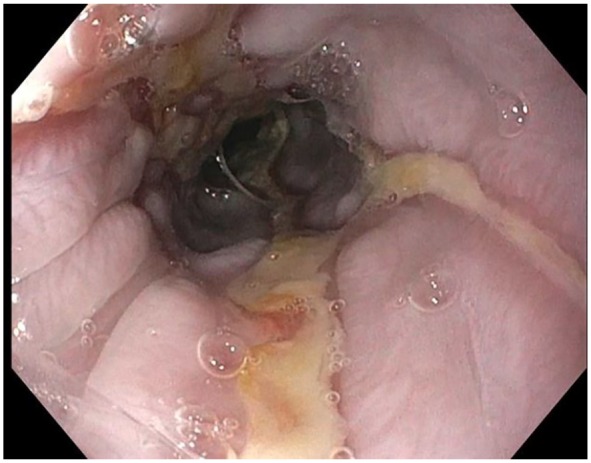
Ulcerations with sloughing mucosa in the distal esophagus.

**Figure 4.**
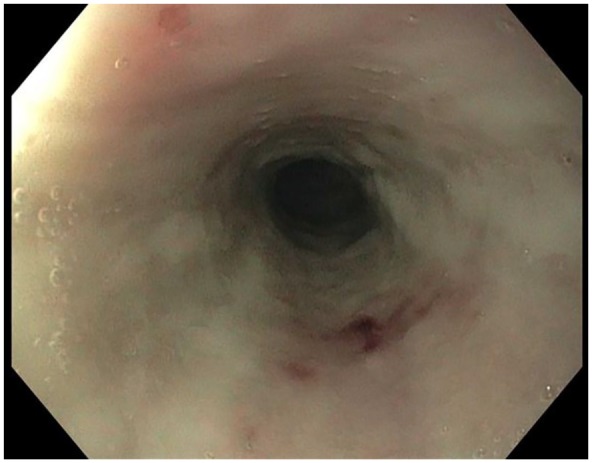
Interval improvement with healing ulceration and decreased sloughing.

On admission, vital signs were normal, and though no major abnormality was noted on physical examination, he was observed coughing up blood-streaked phlegm, small blood clots, and what appeared to be fragments of mucosal tissue. Laboratory studies, including basic metabolic panel and liver function studies, were otherwise within normal limits. Computed tomography scan of his chest showed marked thickening of the distal esophagus. EGD was performed the following day and revealed severe, circumferential esophagitis with deep, serpiginous ulcerations and mucosal sloughing (Figures 2 and 3), most consistent with EDS. Multiple biopsies were taken and showed parakeratosis and minimal inflammation. A thorough skin examination after the procedure was repeated and did not reveal any skin or mucosal blistering or lesions. Direct and indirect immunofluorescence microscopy of the biopsies were negative and no specific immune deposits were present. Infectious etiologies were appropriately ruled out. Further laboratory studies were notable for elevated erythrocyte sedimentation rate (68 mm/h, normal = 0-10 mm/h) and C-reactive peptide (4.5 mg/dL, normal <0.3 mg/dL), normal immunoglobulin G-4 (53.8 mg/dL, normal = 4-86 mg/dL), and serum protein electrophoresis. Given the severity of his symptoms, nonresponse to standard therapies, and no evidence of active infection, he was started on high-dose corticosteroids (intravenous methylprednisolone 125 mg daily for 3 days followed by oral prednisone 40 mg daily for 1 week). He was discharged on prednisone 10 mg daily and omeprazole 40 mg twice daily. His dysphagia and odynophagia subsequently resolved. EGD, 1 month later, demonstrated persistent, but overall much improved esophagitis, without mucosal sloughing or stricture (Figure 4).

## Discussion

Esophagitis dissecans superficialis, also known as “sloughing esophagitis,” is a rare entity first described over 100 years ago.^1^ The clinical presentation varies across a broad spectrum from incidental endoscopic finding to significant disability characterized by profound dysphagia/odynophagia and regurgitation and tends to be more common in the elderly (median age of diagnosis is 65 years).^2^ Expectoration of sloughed, sometimes bloody mucosa can be present, as in our patient. A retrospective study of 21 497 upper endoscopies showed an incidence of EDS of 0.03%.^3^

Esophagitis dissecans superficialis is characterized endoscopically by esophageal inflammation and mucosal sloughing in vertical ribbons.^2^ The necrotic tissue sloughs off in wide, vertical strips. Pathology typically shows necrosis of the superficial layer of the esophageal mucosa leading to separation of this layer from the underlying basal mucosa. Other histologic findings include parakeratosis, basal cell hyperplasia, and focal, minimal inflammation.^4^

A unifying pathogenesis of EDS remains unclear, and most cases are determined to be idiopathic.^3^ However, it has been associated with a variety of exposures and conditions, including malignancy, esophageal trauma, heavy smoking, and pemphigus.^5^ Psychoactive medications, such as SSRIs (selective serotonin reuptake inhibitors), and medications known to cause esophageal irritation, such as bisphosphonates, nonsteroidal anti-inflammatories, and doxycycline, have been implicated.^3,6-8^ EDS appears to occur more commonly in patients with multiple comorbidities, and one study found that 77% of patients with EDS take 5 or more medications,^9^ suggesting an association between EDS and chronic debilitation. Interestingly, there does not seem to be an association with motility disorders or vascular disease, suggesting that local ischemia may not play a role in disease pathogenesis.^9^

The association of EDS and pemphigus, a group of autoimmune disorders causing blistering of skin and mucus membranes, continues to be debated. While both share similar endoscopic and histologic features, most patients with EDS do not circulate typical autoantibodies associated with pemphigus.^2,3^ Most typically, pemphigus involves oropharyngeal mucosa and skin, although there are several case reports of pemphigus vulgaris with exclusively esophageal involvement.^10-12^

Management of EDS is often tailored to the severity of patients’ symptoms or the identification of a modifiable, associated risk factor. In most cases, mucosal sloughing is self-limited and resolves without any long-term sequelae.^2^ Proton pump inhibitors are frequently utilized though their effect seems more to mitigate further injury rather than treat the underlying process.^7^ Immunosuppression maintains a clear role in cases with high probability of or confirmed autoimmune etiology.^11,12^ Though our patient’s presentation was most likely idiopathic, the severity of his presentation and his lack of response to typical, conservative measures prompted us to attempt a trial of high-dose steroids after discussing risks, benefits, and alternatives with the patient. He responded remarkably well in a short period of time, suggesting that steroids may be a viable therapeutic modality to treat severe cases of EDS that fail to respond to typical therapy and raising suspicion for an as-yet unidentified autoimmune component potentially underlying cases of idiopathic EDS.

## Conclusion

Our patient demonstrates a unique case presentation of severe, idiopathic EDS that responded favorably to a short course of high-dose corticosteroids. While EDS remains uncommon, it is paramount that clinicians recognize its endoscopic appearance and potential associations, as it can profoundly affect patients’ quality of life and appropriate clinical management. Specifically, its association with an autoimmune process and whether steroids should be a mainstay of therapy in severe cases warrants further investigation.
